# Expression levels and clinical significance of serum miR-19a/CCL20 in patients with acute cerebral infarction

**DOI:** 10.1515/med-2024-0977

**Published:** 2024-07-02

**Authors:** Yongli Xia, Kun Wei, Lingli Jiang, Dongbo Zou, Yuting Yang, Song Wu, Fei Hu, Yuan Ma

**Affiliations:** Department of Neurosurgery, Affiliated Hospital of Southwest Medical University, Luzhou 646000, Sichuan, China; Clinical Medicine Department, Sichuan College of Traditional Chinese Medicine, Mianyang 621000, Sichuan, China; Department of Neurosurgery, Anzhou District People’s Hospital, Mianyang 622650, Sichuan, China; Department of Neurosurgery, General Hospital of The Western Theater Command, Chengdu 610083, Sichuan, China

**Keywords:** acute cerebral infarction, miR-19a, CCL20, targeted binding, correlation analysis, diagnostic value, ROC curve, combined diagnosis

## Abstract

Acute cerebral infarction (ACI) is a lethal disease whose early diagnosis is critical for treatment. microRNA (miR)-19a targets CC chemokine ligand 20 (CCL20) in myocardial infarction. We investigated the expression patterns of serum miR-19a and CCL20 of ACI patients and assessed their clinical values. Serum samples of 50 healthy subjects and110 ACI patients were collected. Serum levels of miR-19a, CCL20 mRNA, and biochemical indexes were assessed. miR-19a downstream target gene and the binding relationship between miR-19a and CCL20 were predicted and verified. miR-19a and CCL20 mRNA were subjected to correlation and diagnostic efficiency analysis. miR-19a was poorly expressed in the serum of ACI patients, especially in patients with unstable plaque and large infarction. tumor necrosis factor-α, low-density lipoprotein, and platelet/lymphocyte ratio negatively correlated with serum miR-19a level and positively correlated with CCL20. Dual-luciferase assay revealed that miR-19a could negatively regulate CCL20 expression. CCL20 was highly expressed in the serum of ACI patients. The area under receiver-operating characteristic curve of miR-19a combined with CCL20 was 0.9741 (98.00% specificity, 90.91% sensitivity), higher than their single diagnosis. Collectively, miR-19a had high diagnostic value for ACI and could target to restrain CCL20. The combination of miR-19a and CCL20 improved diagnostic value for ACI.

## 1 Introduction

Cerebral ischemia is a medical emergency that results in approximately about 2 million brain cell deaths every minute, necessitating urgent medical treatment [1]. Acute cerebral infarction (ACI) accounts for about 70% of strokes [2]. ACI is a cerebrovascular disease resulting from ischemia, hypoxia, and disrupted cerebral blood circulation, often leading to severe damage in the central nervous system or even death [3,4]. Notably, ACI has a high incidence, mortality, and recurrence rate, resulting in approximately 6.2 million deaths each year worldwide [5]. ACI significantly impacts the quality of life of patients and imposes heavy economic and psychological burdens on families [6]. In the case of any brain diseases, timely diagnosis and treatment are closely associated with morbidity and mortality [7]. Early diagnosis of ACI is critical to its treatment.

microRNAs (miRNAs), small endogenous RNAs, play vital roles as regulators of gene expression [8]. They have emerged as promising therapeutic targets in acute ischemic diseases [9]. For example, plasma miR-409-3p promotes ACI and holds potential for early diagnosis [10]. miR-221 exerts a neuroprotective role in ischemic stroke (IS) by inhibiting pro-inflammatory response [11]. Homo sapiens-miR-19a is downregulated in ACI patients [12]. Silencing miR-19a protects neurons against IS by regulating glucose metabolism and neuronal apoptosis [13]. But, to date, the clinical significance of miR-19a in ACI is less studied.

CC chemokine ligand 20 (CCL20), also known as liver and activation-regulated chemokine, Exodus-1, and macrophage inflammatory protein-3α, is part of the subfamily of small CC cytokine [14, 15]. In normal and stable states, CCL20 is expressed in various epithelial cells [16, 17] and immune cells [15, 18]. In response to various inflammatory conditions, CCL20 is typically upregulated, contributing to the inflammatory cascade reaction [19, 20]. However, whether miR-19a and CCL20 can serve as clinical biomarkers of ACI, thus providing potential diagnostic value for ACI, remains unknown. The purpose of this study was to explore the clinical meaning and underlying regulatory effect of miR-19a and CLL20 by examining their expression patterns in the serum of ACI patients, with the goal of offering reference value for the early diagnosis of ACI.

## 2 Material and methods

### 2.1 Sample size estimation

Sample size estimation was carried out using the sample size estimation software G Power 3.0.10 (University of Düsseldorf, Nordrhein-Westfalen, Germany), with the corresponding parameters set as Power = 0.80, *α* = 0.05, and the ratio *N*2:*N*1 = 1:2. The assessment found that the minimum total score was 144, the minimum number of people for N1 was 96, and the minimum number of people for N2 was 48 (Figure S1: G Power Sample size estimation).

### 2.2 Study subjects

A total of 110 ACI patients hospitalized at the Affiliated Hospital of Southwest Medical University within 24 h after the attack between May 2016 and May 2017 were consecutively recruited. In addition, 50 cases of unrelated individuals were selected from the health examination center of the Affiliated Hospital of Southwest Medical University as the healthy control group. Demographic information including age, sex and body mass index (BMI) was documented.


**Ethical approval:** This study was approved by the Ethics Committee of Affiliated Hospital of Southwest Medical University (no. 2022EC4-ky066).
**Consent to participate statement:** All participants provided written informed consent prior to enrollment.

### 2.3 Inclusion and exclusion criteria

Inclusion criteria for the ACI group were as follows: [1] diagnosed as ACI based on the clinical diagnosis of ACI in the Chinese Guidelines for the Diagnosis and Treatment of Acute IS; [2] within 24 h of the onset; [3] age >18 years; [4] confirmed as ACI triggered by intracranial artery system infarction through computed tomography (CT) or magnetic resonance imaging and immediately received standardized treatment (including anticoagulant or anti-platelet medicine); [5] underwent intravenous thrombolysis or mechanical thrombolysis within 24 h of onset.

Exclusion criteria for the ACI group were as follows: [1] malignant neoplastic diseases; [2] severe hepatic and renal dysfunctions; [3] intracranial hemorrhage; [4] acute infarction over 24 h [5] acute infectious diseases; [6] a history of surgical treatment or trauma in the past 3 months; [7] complication of myocardial infarction; [8] a history of use of statin-based lipid-lowering drugs, antiplatelet drugs, or angiotensin-converting enzyme inhibitor cumulative drugs in the past 6 months; [9] a previous history of stroke; [10] could not undergo thrombolysis or thrombectomy.

Inclusion criteria for the control group included normal indicators, age >18 years, and no infectious diseases.

The following were the exclusion criteria for the control group: [1] a history of surgical treatment or trauma in the past 3 months; [2] a history of use of statin-based lipid-lowering drugs, antiplatelet drugs, or angiotensin-converting enzyme inhibitor cumulative drugs in the past 6 months; [3] a previous history of stroke; [4] a history of Parkinson’s disease; [5] a history of acute infections in the past 3 months; [6] a history of coronary heart disease.

### 2.4 Assessment of disease

(1) ACI patients were categorized into types I–VI corresponding to different states of plaque lesions according to the classification method of the American Heart Association (AHA) [21], among which types I–III referred to stable plaque (stable plaque/early stage of lesion) and IV–VI referred to unstable plaque.
(2) Referring to the Adama typing method [21] and the content of the Guangzhou National Symposium on Cerebrovascular Diseases Stroke Classification and Staging Treatment (Draft Recommendation) in 2000, the size of cerebral infarction was assigned into large infarction (L, over 1 lobe of brain, >5.0 cm), medium infarction (M, less than 1 lobe of brain, 3.1–5.0 cm), small infarction (S, 1.6–3.0 cm), lacunar infarction (LI, <1.5 cm), and multiple infarction (Mu, multiple medium, small, and lacunar infarction) based on the CT classification.
(3) ACI patients were classified based on the following infarct localization: basal ganglia infarction (BGI) (contralateral hemiplegia, including central facial and tongue palsy, contralateral hemiparesis and even hemianopsia if the internal capsule is involved), frontal lobe infarction (FLI) [dystaxia, mental disorder and emotional abnormality, accompanied by motor aphasia (oral expression disorder was the most obvious, mainly manifested as non-fluent communication, telegraphic language, laborious speech, difficulty in finding words, inappropriate use of words, relatively limited oral comprehension ability)], temporal lobe infarction (TLI) [mental disorder, poor learning ability, deterioration of the memory, accompanied by sensory aphasia (listening-comprehension disorder was the most obvious, mainly manifested as excessive and chaotic speech, off topic answers, difficulty in understanding, usually accompanied by reading and writing impairments)], cerebellar infarction (CI) (dizziness, walking instability, dystaxia, language disorder and ipsilateral decrease of muscle tone), brainstem infarction (BI) (typically needle-like pupils, high fever, coma and tetraplegia, and mild BI manifests with hemiplegia, sensory disturbance and bulbar paralysis).
(4) Classification according to the Trial of Org 10172 in Acute Stroke Treatment (TOAST) [22] included small vessel occlusion (SVO), large artery atherosclerosis (LAA), cardioembolism (CE), stroke of other determined etiologies (SOE), and stroke of undetermined etiology (SUE).
(5) The severity of stroke was determined using the National Institutes of Health Stroke Scale (NIHSS) score, a 15-item tool for nervous system assessment [23]. Therefore, ACI patients were classified to severe cerebral infarct (SCI) group (score >15), moderate cerebral infarct (MOCI) group (8 < scores < 15), and mild cerebral infarct (MICI) group (scores ≤8).
(6) The stenosis of blood vessels was evaluated by color Doppler ultrasonic examination, and patients with ≥50% stenosis were recorded in accordance with the criteria of the North American Symptomatic Carotid Endarterectomy Trial [24] and past research [25].

### 2.5 Blood collection and biochemical examination

Blood sample was collected from ACI patients immediately upon admission, and that of healthy controls was collected once immediately after recruitment. Blood sample (5 mL) was collected from each participant via the median cubital vein into a 10.0 mL 150 USP-unit heparin sodium anticoagulant tube (green, 367874, BD Biosciences, Franklin Lakes, NJ, USA). Thereafter, the blood samples were centrifuged at 1,000×*g* for 10 min, and the serum was stored in the eppendorf tubes at −80°C for subsequent analysis. Platelets and lymphocytes in whole blood were counted, and platelet/lymphocyte ratio (PLR) was automatically calculated using the Sysmex XE-5000 automatic hematology system (Sysmex, Kobe, Japan). Blood biochemical examination was conducted to measure total cholesterol (TC), triglyceride (TG), low-density lipoprotein (LDL), high-density lipoprotein (DHL), homocysteine (Hcy), and human inflammatory factor tumor necrosis factor-α (TNF-α) using corresponding human TC (ab65390), TG (ab65336), LDL (ab270212), HDL (ab125961), Hcy (ab228559), and human inflammatory TNF-α (ab178013) kits (Abcam, Cambridge, MA, USA). Then, 10 μL of serum sample and 40 μL of sample diluents were added into each well of the enzyme-coated plates. After sealing, the plates were incubated in a warm bath at 37°C for 30 min. After 5 washes, 50 μL of enzyme labeling reagent was added into the reaction plates, which were then sealed and incubated in a warm bath at 37°C for another 30 min. Next, the reactive plates were added with 50 μL of chromophoric reagent A and 50 μL of chromophoric reagent B, mixed, and placed at 37°C in the absence of light for 15 min to allow color development. To terminate the reaction, each well was added with 50 μL termination solutions, and the color changed from blue to yellow by the time. Absorbance at 450 nm was detected using a Multiskan™ FC microplate reader (Thermo Fisher Scientific). The levels of related factors were calculated using standard curve.

### 2.6 Total RNA extraction

The levels of serum miR-19a and CCL20 mRNA were determined by reverse transcription-quantitative polymerase chain reaction (RT-qPCR). Total RNA of blood serum was extracted using RNAzol (Invitrogen; Thermo Fisher Scientific, Inc., Waltham, MA, USA). Initially, 400 μL of the sample was combined with 1 mL RNAzol in a 1.5 mL centrifugation tube, evenly mixed by shaking, and allowed to stand for 5 min. Subsequently, 400 μL of ddH_2_O was added to every 1 mL RNAzol, mixed for 15 s by shaking, incubated for 10 min at room temperature and centrifuged at 10,000*g* for 15 min. The supernatant was collected and placed in a new 1.5 mL centrifugation tube, added with an equal amount of isopropanol, incubated for 10 min at room temperature, and centrifuged at 10,000*g* for 10 min to eliminate the supernatant. The precipitation was added with 400 μL of 75% ethanol, mixed, and centrifuged for 3 min at 7,000*g* (this procedure was repeated once). The supernatant was discarded, and the precipitation was naturally dried. Then, 50 μL of ddH_2_O (RNase and DNase-free) was added and dissolved to obtain the total RNA.

### 2.7 RT-qPCR

RNA concentration and purity were assessed using a spectrophotometer (QuickDrop, Meigu Molecular Instruments, Shanghai, China). RNA was reverse-transcribed into cDNA utilizing the cDNA reverse transcription system (Thermo Fisher Scientific) following the provided instructions. Quantitative PCR amplification was conducted using the SYBR^®^ Premix Ex Taq^TM^ kit (Takara Bio, Inc., Otsu, Japan) in strict accordance with the provided instructions, with U6 or GAPDH serving as internal controls. The specific program was 95°C, 30 s, followed by 40 cycles of 95°C, 10 s and 60°C, 30 s. Relative expression was determined using the 2^-ΔΔCt^ method [26]. Each sample was analyzed independently, and all experiments were repeated three times. PCR primers are illustrated in Table 1.

**Table 1:** RT-qPCR primer sequences

Name of primer	Sequences
miR-19a-F	GCGTGGCAATCTATGCAA
miR-19a-3p-R	GTGCAGGGGTCCGAGGT
CCL20-F	ATGTGCTGTA CCAAGAGTTTG
CCL20-R	GGAGTAGCAGCACTGACATCA
U6-F	CTCGCTTCGGCAGCACATATACTA
U6-R	ACGAATTTGCGTGTCATCCTTGCG
GAPDH-F	CATCACCATCTTCCAGGAGCG
GAPDH-R	TGACCTTGCCCACAGCCTTG

Note: miR, microRNA; F, forward; R, reverse; CCL20, CC chemokine ligand 20; GAPDH, glyceraldehyde-3-phosphate dehydrogenase.

### 2.8 Bioinformatic analysis

The downstream target genes of miR-19a were predicted through Starbase website (http://starbase.sysu.edu.cn/), RNAInter website (http://www.rna-society.org/raid/search.html), and Jefferson website (https://cm.jefferson.edu/rna22/Precomputed/), and the intersection was taken. The binding sites of miR-19a and CCL20 were predicted through Starbase website.

### 2.9 Dual-luciferase reporter assay

The wild-type (CCL20-wt) and mutant (CCL20-mut) luciferase plasmids were constructed by cloning the binding and mutant sequences onto the luciferase vectors pGL3 (Pro-mega, Madison, WI, USA). The 293T cells (ATCC, Manassas, VA, USA) were seeded in 6-well plates (2 × 10^5^ cells/well) and cultured for 24 h. Lipofectamine 2000 (11668-019, Invitrogen, Carlsbad, CA, USA) was used to introduce the constructed luciferase vectors into 293 T cells alongside mimic-NC or miR-19a mimic (Genechem, Shanghai, China) (miRNA-mimic 50 nM). Following 24 h transfection, the luciferase activity was evaluated using the Dual-Lucy Assay kit (Solarbio, Beijing, China). Cell experiment was independently replicated three times.

### 2.10 Statistical analysis

SPSS 21.0 (IBM Corp., Armonk, NY, USA) and GraphPad Prism 8 (GraphPad Software, San Diego, CA, USA) were used for data analysis and graphing. The normal distribution of data was tested using Shapiro–Wilk and Kolmogorov–Smirnov tests. Normally distributed measurement data were presented as mean ± standard deviation, with comparisons between groups made using the independent sample *t* test. Non-normally distributed measurement data were expressed as quartiles [median (maximum, minimum)], with comparisons between groups conducted using the Mann–Whitney *U* test, and those among groups using the Kruskal–Wallis rank sum test, followed by Tukey’s multiple comparison test. Counting data were represented as cases and percentage, with the chi-square test used. Comparative analysis of categorical variables was performed using Fisher’s exact test. The diagnostic value of serum miR-19a and CCL220 in ACI was evaluated using receiver-operating characteristic (ROC) curve. The correlation among measurement data was analyzed using Spearman’s correlation analysis. The combined diagnostic factor of the two markers was analyzed using binary logistic regression. A value of *P* < 0.05 was indicative of statistical significance.


**Ethics statement:** This study was conducted with approval of the Ethics Committee of Affiliated Hospital of Southwest Medical University. All participants provided written informed consent prior to enrollment.

## 3 Results

### 3.1 Clinical features of patients

This study included 110 ACI patients and 50 healthy subjects. Clinical data are illustrated in Table 2, showing no statistically significant differences in age and sex between the two groups (*P* > 0.05). However, the differences of BMI, TC, TG, LDL, HDL, Hcy, PLR, and TNF-α were significant (all *P* < 0.05). All indexes except HDL were higher in the ACI group than those in the control group.

**Table 2:** Clinical data of patients and controls

		ACI (*n* = 110)	Control (*n* = 50)	*X* ^2^/*t*	*P*
Age (years)		61.54 ± 7.61	60.22 ± 9.53	1.14	0.26
Sex (Male/Female)		77/33	38/12	0.61	0.43
AHA classification	unstable plaque	42 (38.18%)	—	—	—
	stable plaque	68 (61.82%)	—	—	—
Infarct size	L	14 (12.73%)	—	—	—
	M	33 (30.00%)	—	—	—
	S	36 (32.73%)	—	—	—
	LI	19 (17.27%)	—	—	—
	Mu	8 (7.27%)	—	—	—
Infarct localization	BGI	64 (58.18%)	—	—	—
	FLI	19 (17.27%)	—	—	—
	TLI	14 (12.73%)	—	—	—
	CI	8 (7.27%)	—	—	—
	BI	5 (4.55%)	—	—	—
TOAST type	SVO	44 (40.00%)	—	—	—
	LAA	30 (27.27%)	—	—	—
	CE	21 (19.09%)	—	—	—
	SOE	9 (8.18%)	—	—	—
	SUE	6 (5.46%)	—	—	—
NIHSS	SCI	12 (10.91%)	—	—	—
	MOCI	61 (55.45%)	—	—	—
	MICI	37 (33.64%)	—	—	—
Ipsilateral carotid stenosis ≥50%		23 (20.91%)	—	—	—
Contralateral carotid stenosis ≥50%		16 (14.55%)	—	—	—
Vertebral artery stenosis ≥50%		3 (2.73%)	—	—	—
BMI		23.61 ± 2.56	19.22 ± 2.19	10.5	<0.001
TC (mmoL/L)		4.35 ± 0.39	3.77 ± 0.54	7.69	<0.001
TG (mmoL/L)		1.54 ± 0.35	0.92 ± 0.28	11.02	<0.001
LDL (mmoL/L)		3.10 ± 0.62	2.52 ± 0.71	5.24	<0.001
HDL (mmoL/L)		1.02 ± 0.43	1.41 ± 0.33	5.69	<0.001
Hcy (mmoL/L)		15.43 ± 4.91	12.77 ± 2.48	3.62	<0.001
TNF-a (pg/mL)		132.60 ± 10.94	75.53 ± 10.64	30.85	< 0.001
PLR		220.71 ± 71.21	120.39 ± 36.92	9.39	< 0.001

Note: ACI, acute cerebral infarction; L, large; M, medium; S, small; LI, lacunar infarction; BGI, basal ganglia infarction; FLI, frontal lobe infarction; TLI, temporal lobe infarction; CI, cerebellar infarction; BI, brainstem infarction; TOAST, Trial of Org 10172 in Acute Stroke Treatment; SVO, small vessel occlusion; LAA, large artery atherosclerosis; CE, cardioembolism; SOE, stroke of other determined etiologies; SUE, stroke of undetermined etiology; NIHSS, National Institutes of Health Stroke Scale; MICI, mild cerebral infarct; MOCI, moderate cerebral infarct; SCI, severe cerebral infarct; BMI, body mass index; TC, total cholesterol; TG, triglyceride; LDL, low density lipoprotein; HDL, high density lipoprotein; Hcy, homocysteine; TNF-a, tumor necrosis factor-α; PLR, platelet/lymphocyte ratio. Normally distributed measurements were denoted as mean ± standard deviation, and comparisons between groups were made using the independent sample *t* test. Counting data were expressed as the number of cases (percentage) and tested by the chi-square test, and comparative analyses of categorical variables were conducted using Fisher’s exact test.

### 3.2 Low expression of serum miR-19a had auxiliary diagnostic values on ACI

**Figure 1:**
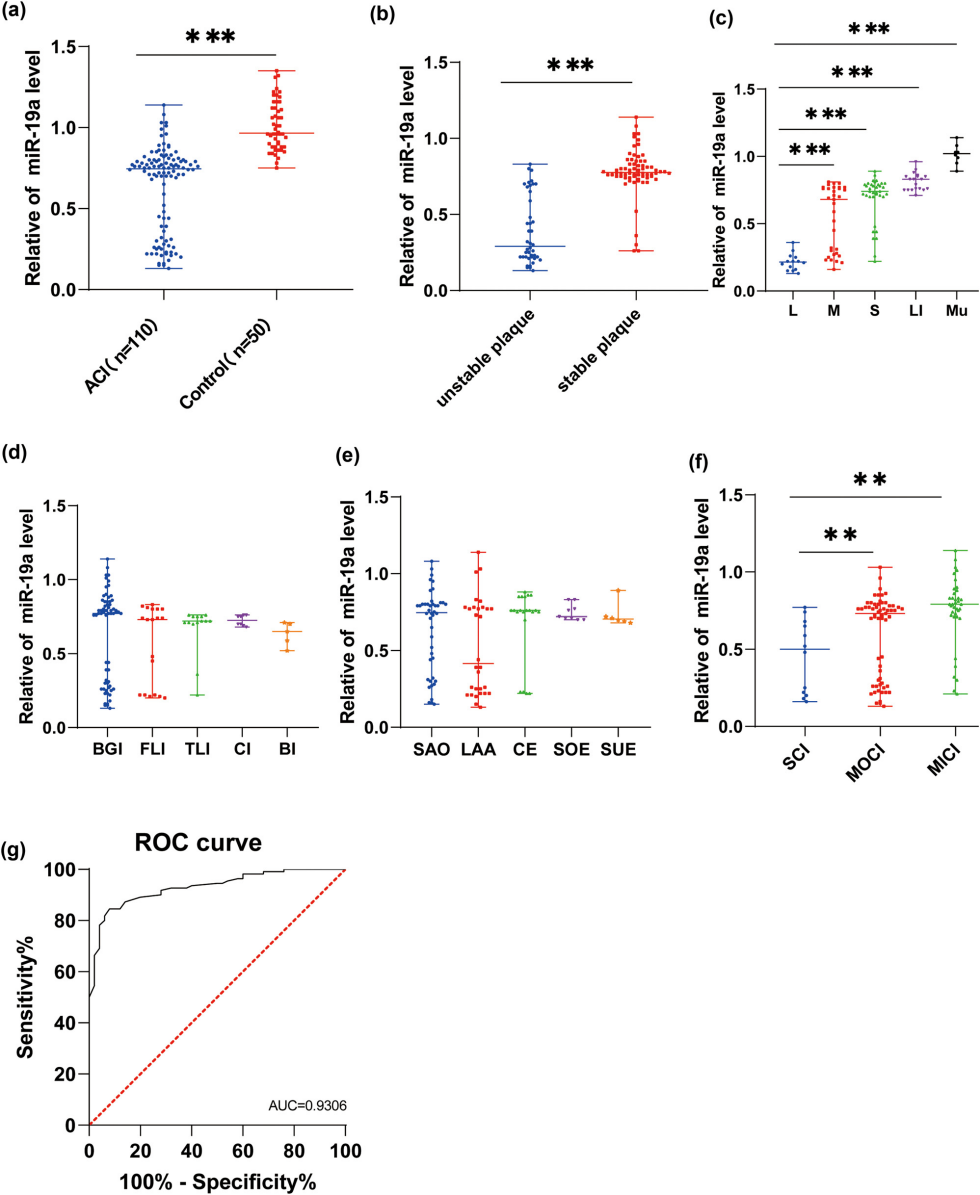
Weak expression of serum miR-19a had auxiliary diagnostic value on ACI: (a) miR-19a expression in the serum of ACI patients and healthy controls was detected by RT-qPCR; (b) miR-19a expression in the unstable plaque group and the stable plaque group was detected; (c): miR-19a expression in serum of ACI patients in large infarction group (L), medium infarction group (M), small infarction group (S), lacunar infarction group (LI), and multiple infarction group (Mu) was detected; (d) miR-19a expression in serum of patients in the BGI, FLI, TLI, CI, and BI groups was determined; (e) serum miR-19a expression in serum of ACI patients with SVO, LAA, CE, SOE, and SUE was determined; (f) miR-19a expression in serum of ACI patients in the SCI, MOCI, and MICI groups was measured; (g) ROC curve of serum miR-19a in ACI. Normal distribution was examined by Shapiro–Wilk and Kolmogorov–Smirnov tests. Measurement data conforming to non-normal distribution were presented by quartiles, which were median values (maximum, minimum). Mann–Whitney *U* test was adopted for comparisons between two groups, while the Kruskal–Wallis rank sum test was applied for comparisons among multiple groups, followed by Tukey’s test. **P < 0.01, ***P < 0.001.

Serum miR-19a expression levels in both healthy controls and ACI patients was detected by RT-qPCR (Figure 1a). The result showed that the expression of serum miR-19a was decreased in ACI group (*P* < 0.001). Based on AHA classification, ACI patients were assigned into the unstable plaque group (*n* = 42) and the stable plaque group (*n* = 68). The result showed that miR-19a was poorly expressed in the unstable plaque group relative to that in the stable plaque group (all *P* < 0.001, Figure 1b). Moreover, ACI patients were categorized into five groups based on cerebral infarction size: the large infarction group (L, *n* = 14), medium infarction group (M, *n* = 33), small infarction group (S, *n* = 36), lacunar infarction group (LI, *n* = 19), and multiple infarction (Mu, *n* = 8). The findings demonstrated a predominant decrease in miR-19a expression, particularly in the large infarction group (all *P* < 0.001, Figure 1c). No significant difference in miR-19a expression was identified between patients with different infarct localization and TOAST classifications (Figure 1d and e, all *P* > 0.05). Furthermore, miR-19a expression was substantially increased in the MICI group relative to that in the MOCI and SCI groups, as per NIHSS (Figure 1f, all *P* < 0.01). Additionally, the ROC curve of serum miR-19a level in ACI diagnosis showed that the area under the curve was 0.9306 with 0.835 cut-off value, 92.00% specificity, and 84.55% sensitivity, indicating that a serum miR-19a level <0.835 could aid in the diagnosis of ACI (Figure 1g).

### 3.3 miR-19a level was negatively correlated with TNF-α, LDL, and PLR

**Figure 2:**
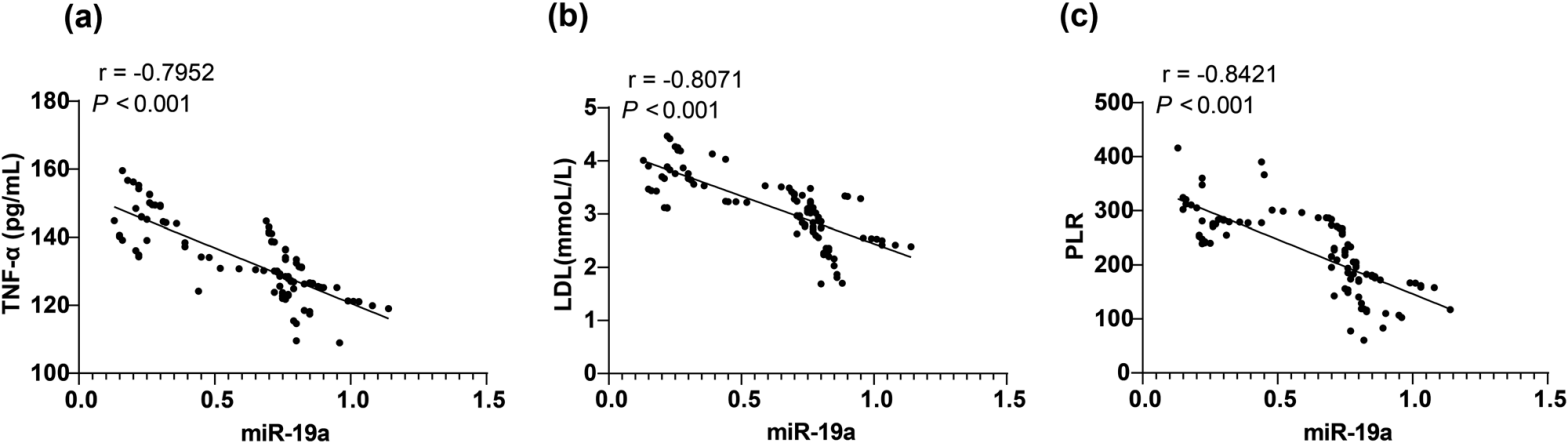
Correlation analyses of miR-19a with TNF-α, LDL, and PLR in the serum of patients with ACI. The correlation between miR-19a and blood indexes in the serum of ACI patients was analyzed: (a) miR-19a was negatively correlated with TNF-α serum level, (b) miR-19a was negatively correlated with LDL serum level, and (c) miR-19a was negatively correlated with PLR. All data were counting data and analyzed using Spearman’s correlation analysis.

The correlation between serum miR-19a and PLR, serum lipids, Hcy, and other pro-inflammatory factors was analyzed by calculating Spearman’s coefficients. The results showed that miR-19a was negatively correlated with TNF-α, LDL, and PLR (with *r*-values of −0.7952, −0.8071, and −0.8421, respectively, all *P* < 0.001, Figure 2a–c). However, there was no obvious correlation between miR-19a and TG, TC, HDL, and Hcy indexes (Table S1, *P >* 0.05).

### 3.4 CCL20 expression was increased in the serum of ACI patients

**Figure 3:**
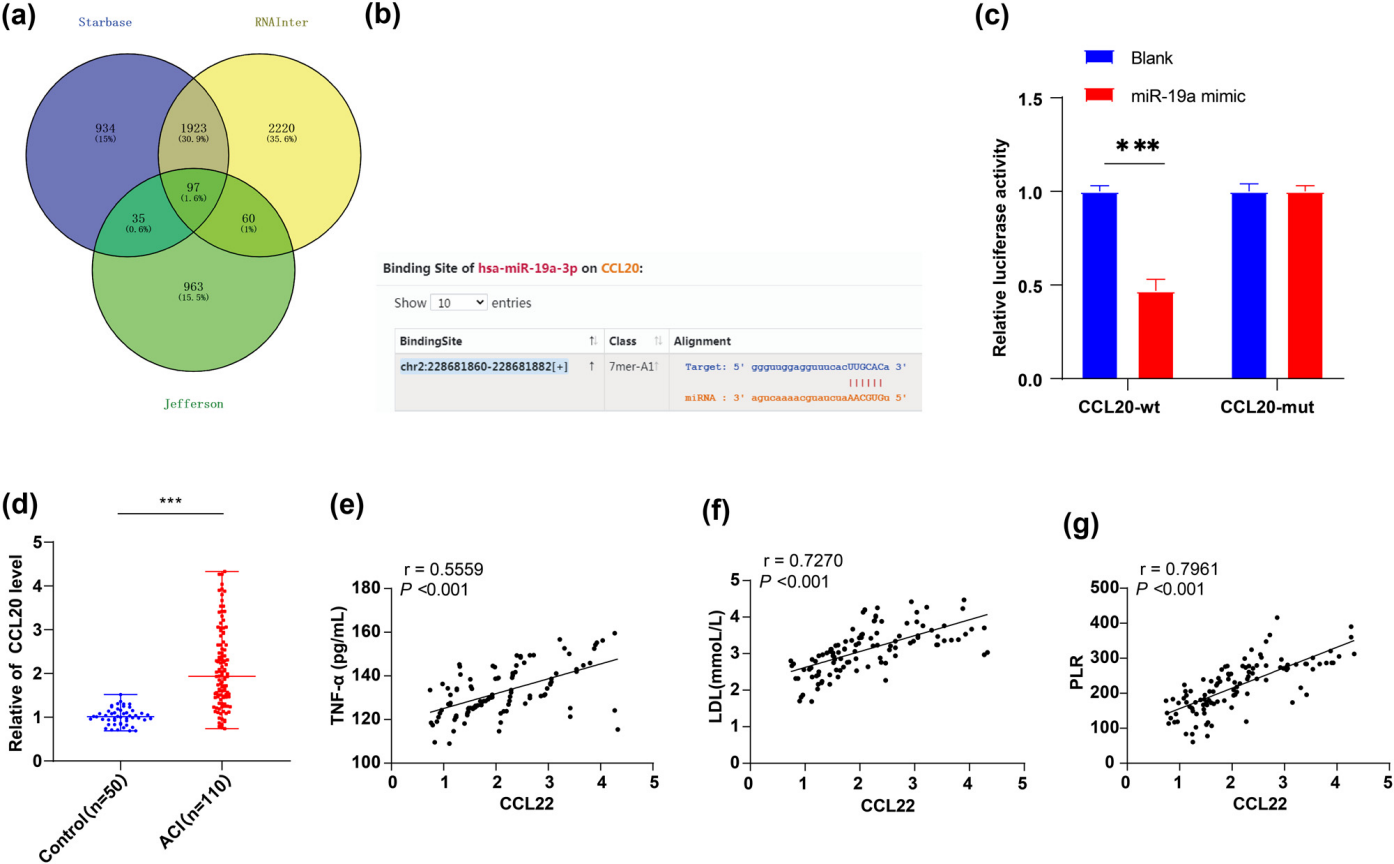
miR-19a targeted to negatively regulate CCL20 expression in the serum of ACI patients: (a) the downstream target genes of miR-19a were predicted through Starbase website, RNAInter website, and Jefferson website, and intersection was taken; (b) the binding sites of miR-19a and CCL20 were predicted on Starbase website; (c) dual-luciferase reporter assay; (d) serum CCL20 mRNA expression was detected by RT-qPCR; (e–g) correlation of CCL20 with TNF-α (e), LDL (f), and PLR (g) was analyzed by Spearman s correlation analysis. Data in panel c were counting data. All data were analyzed using *t* test. ***P < 0. 001.

In our quest to unravel the functional mechanism of miR-19a in ACI, we employed the Starbase website (http://starbase.sysu.edu.cn/), RNAInter website (http://www.rna-society.org/raid/search.html), and Jefferson website (https://cm.jefferson.edu/rna22/Precomputed/) to predict downstream target genes of miR-19a, culminating in the identification of an intersection (Figure 3a). Attention was paid to chemokine CCL20 because a previous study has showed that CCL20 often plays a pro-inflammatory role in the brain [27]. Also, a previous study has demonstrated that miR-19a represses the expression of CCL20, and miR-19a directly targets CCL20 and negatively manipulate its expression [28]. Therefore, the binding sites of miR-19a and CCL20 were predicted on Starbase website (Figure 3b). Furthermore, dual-luciferase reporter assay revealed that miR-19a negatively modulated CCL20 expression in 293 T cells (Figure 3c), affirming a target relationship between miR-19a and CCL20. Furthermore, RT-qPCR showed that CCL20 expression was significantly increased in the serum of ACI patients (*P* < 0. 001, Figure 3d). Spearman’s correlation analysis revealed positive correlations between CCL20 and TNF-α, LDL, and PLR (the r-values were 0.5559, 0.7270, and 0.7961, separately, Figure 3e–g, all *P* < 0.001), while no associations were observed between CCL20 and TG, TC, HDL, and Hcy (Table S2, *P* > 0.05).

### 3.5 Combination of serum miR-19a and CCL20 increased the diagnostic value on ACI

**Figure 4:**
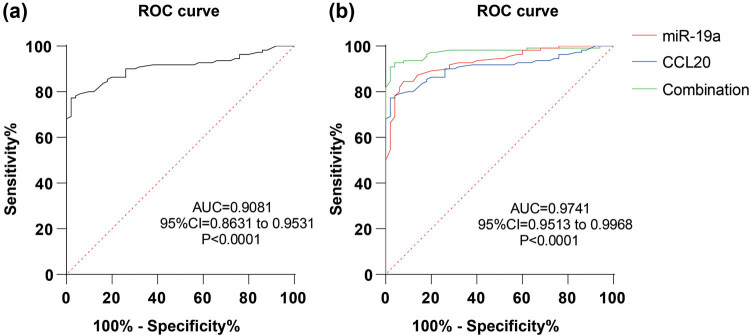
Combination of serum miR-19a and CCL20 increased the diagnostic value on ACI: (a) ROC curve analysis of serum CCL20 on ACI diagnosis and (b) ROC curve analysis of serum miR-19a combined with CCL20 on ACI diagnosis.

Based on the aforementioned results, the diagnostic value of serum CCL20 in ACI was analyzed using ROC curve analysis. The area under the curve was 0.9081 with 1.380 cut-off value, 98.00% specificity, and 77.27% sensitivity (Figure 4a). Then, the diagnostic value of miR-19a combined with CCL20 on ACI was analyzed. The area under the curve was 0.9741 with 98.00% specificity and 90.91% sensitivity (Figure 4b). These results suggest that the combination of serum miR-19a and CCL20 offers higher diagnostic efficacy for ACI than miR-19a or CCL20 alone.

## 4 Discussion

ACI represents a type of cardiovascular disease characterized by thrombosis of cerebral blood supply artery, atherosclerotic or allopathic flow into the carotid artery and cerebral artery, resulting in brain tissue necrosis or softening. Additionally, approximately 1% of cerebral infarctions are attributed to hematological diseases involving the red blood cells, white blood cells, platelets, and pro-coagulant [29], ultimately posing a huge threat to human health and the safety of life [30,31]. The CCL chemokine family has long been recognized as an important contributor to inflammation in diseases, with its rebalance playing a pivotal role in maintaining homeostasis and protecting the central nervous system [32]. Evidence suggests that serum CCL23 expression holds certain diagnostic value on ACI [33], while CCL20 is identified as a potential target in IS [34]. Consequently, we directed our attention to CCL20 and further located its upstream regulator miR-19a. miRNAs play an essential role in neural development and function [35]. Increasing evidence has found differential expressions of miRNAs in the serum of ACI patients [36,37]. This study found that miR-19a was significantly downregulated in ACI patients. The expression patterns of miR-19a in previous studies are shown in Table S3.

A previous study has reported that miR-19a expression is decreased in ACI [12]. After ACI classification, the results showed that miR-19a expression in patients with unstable plaque was lower than that in patients with stable plaque. Carotid plaque is clinically related to the degree of neural function deficits in ACI patients [38]. Clinically, the stability of carotid plaque is often associated with atherosclerosis and the cardiovascular diseases and atherosclerosis is perceived as a major cause of ACI [39]. Meanwhile, the results showed that miR-19a expression was decreased with the increase of cerebral infarct size and was increased in MICI patients relative to that in MOCI and SCI patients.

The ROC curve holds extensive utility in the evaluation of medical diagnostic value [40]. In our study, the ROC curve of serum miR-19a in ACI diagnosis showed that the area under the curve was 0.9306 with 0.835 cut-off value, 92.00% specificity, and 84.55% sensitivity. miR-19a is considered as a promising novel indicator of early diagnosis of acute myocardial infarction [41]. Thus, weak expression pattern of serum miR-19a possessed auxiliary diagnostic value on ACI. Inflammation is a key factor in ACI [42]. fInflammation is a basic pathogenic factor during the development of atherosclerosis, and TNF-α is a key pro-inflammatory cytokine involved in the inflammatory reaction and plays a pivotal role in the formation and development of atherosclerotic plaque [43]. Researchers believe that ACI pathogenesis is attributed to lipid deposition, mainly LDL deposition in the cerebral arterial wall, arterial intimal thickening, luminal stenosis, which affects blood supply of corresponding brain tissues, and finally bringing about ischemic and hypoxic necrosis [44]. In addition, mounting evidence suggests that PLR can be used as a potential clinical marker of ACI [45,46]. More specifically, high PLR is a reflection of the burden of plaques with high risk [47]. In our study, we found that serum miR-19a expression was negatively associated with TNF-α, LDL, and PLR. Weak miR-19a expression may correlate with inflammation in ACI.

To explore the downstream mechanism of miR-19a in ACI, we predicted its downstream target genes of miR-19a and identified intersections. CCL20 could play a role as pro-inflammatory chemokine in the brain [27]. The binding sites of miR-19a and CCL20 were predicted, indicating a potential target relation between miR-19a and CCL20. The T-cell infiltration into the brain after IS is driven by the chemokine CCL20 [48]. CCL20 expression was elevated in the retinal ganglion cells of repetitive traumatic brain injury [49]. The result showed that CCL20 expression was increased in the serum of ACI patients. The expression of CCL20 can be elevated by TNF-α and IL-17A [14]. LDL induces CCL20 expression in vascular smooth muscle cells in a dose- and time-reliant way [50]. Anti-CCL20 can reduce neutrophil and platelet accumulation [17]. Our results indicated positive correlations between CCL20 and TNF-α, LDL, and PLR. Furthermore, miR-19a has been found to attenuate hypoxia-reoxygenation-induced injury by suppressing CCL20 in human embryonic cardiomyocytes [28]. In rats subjected to lateral fluid percussive impact, up-regulated CCL20 expression accompanied neurodegenerative injury, suggesting a role for CCL20 in progressive neurodegenerative injury [51]. The ROC curve of serum CCL20 in our study showed that the area under the curve was 0.9081 with 1.380 cut-off value, 98.00% specificity, and 77.27% sensitivity. ROC curve of serum miR-19a combined with CCL20 showed that the area under the curve was 0.9741 with 98.00% specificity and 90.91% sensitivity. These results indicate that serum miR-19a combined with CCL20 offers higher diagnostic efficacy for ACI compared to miR-19a or CCL20 alone. The expression patterns of CCL20 in previous studies are presented in Table S4.

In conclusion, this study revealed that the combination of serum miR-19a and CCL20 increased the diagnostic value on ACI and offered a probability for clinical ACI diagnosis via the combination of miR-19a and CCL20. This study hypothesized that miR-19a played a role in ACI by regulating inflammatory factors based on previous studies. However, the purpose of this study was to explore ACI induced by anterior circulation, and study subjects were patients with ACI induced by intracranial artery system infarction, while posterior circulation that can also trigger infarction was not studied. The area of infarction caused by vascular occlusions may involve the cerebral cortex and subcortical areas, brainstem, cerebellum, internal capsule, and semi-oval centers. After dividing ACI patients to the BGI, FLI, TLI, CI, and BI groups, we found no statistical significance in miR-19a expression among groups. We only scratched the surface of the correlation of miR-19a with infarct size and serum indicators, which only indicates indirectly that serum-related indicators may affect infarct size, but we have not validated it further. What is more, this study only focused on the stability of carotid plaque, and the ways by which carotid plaques evoke infarction may also vary. The carotid plaque may fall off, thus leading to insufficient blood supply to distal tissues, or severe stenosis of the intracranial artery may occur due to the enlargement of the plaque. Finally, this study simply revealed a target relation between miR-19a and pro-inflammatory chemokine CCL20. The deeper molecular mechanisms were not explored. This study did not measure biomarkers on several occasions, upon admission, after 24 and 48 h and followed the dynamics, which reduced the interestingness of the study. Future studies should pay attention to the underlying mechanism of miR-19a in ACI from the perspective of the molecular mechanism of miR-19a targeting CCL20, and comprehensive analyses in the interference of thrombolysis on miR-19a in ACI patients and the changes of miR-19a in ACI patients in different periods after admission for treatment, so as to deeply investigate the clinical significance of miR-19a expression in ACI patients.

## Supplementary Material

Supplementary material
