# Supplementary material for: Expression levels and clinical significance of serum miR-19a/CCL20 in patients with acute cerebral infarction
Source: Open Med (Wars). 2024 Jul 2;19(1):20240977. doi: 10.1515/med-2024-0977 (PMC11221218; doi:10.1515/med-2024-0977)
Supplement: Supplementary material [file med-2024-0977-sm.pdf]

Supplementary material

Table S1: Correlation coefficient of miR-19a with TG, TC, HDL and Hcy

	r	*P*
TG	0.1249	0.1937
TC	0.0539	0.5758
HDL	0.0515	0.5933
Hcy	−0.0502	0.6024

Note: miR, microRNA; TG, triglyceride; TC, total cholesterol; HDL, high density lipoprotein; Hcy, homocysteine.

Table S2: Correlation coefficient of CCL20 with TG, TC, HDL and Hcy

	r	*P*
TG	−0.1253	0.1923
TC	−0.0474	0.6233
HDL	−0.0400	0.6777
Hcy	0.0517	0.5917

Note: CCL20, CC chemokine ligand 20; TG, triglyceride; TC, total cholesterol; HDL, high density lipoprotein; Hcy, homocysteine.

Table S3: Expression of miR-19a in previous studies

Expression patterns	Diseases	Authors
Decrease	Myocardial infarction	Qiang Fu et al. (PMID: 33165673)
Decrease	Ischemic stroke (neurons)	Xiao-Li Ge et al. (PMID: 31168302)
Increase	Ischemic stroke (astrocytes)	Xiao-Li Ge et al. (PMID: 31168302)
Increase	Acute myocardial infarction	Fatemeh Mansouri et al. (PMID: 32334458)

Note: miR, microRNA.

Table S4: Expression of CCL20 in previous studies

Expression patterns	Diseases	Authors
Increase	Various tumors	Weilong Chen et al. (PMID: 32060846)
Increase	Myocardial infarction	Qiang Fu et al. (PMID: 33165673)
Increase	Neuroinflammation	Minako Ito et al. (PMID: 30602786)
Increase	Neurodegeneration	Christopher C Leonardo et al. (PMID: 24323811)
Increase	Neuroinflammation	Gordon P Meares et al. (PMID: 22319003)

Note: CCL20, CC chemokine ligand 20.

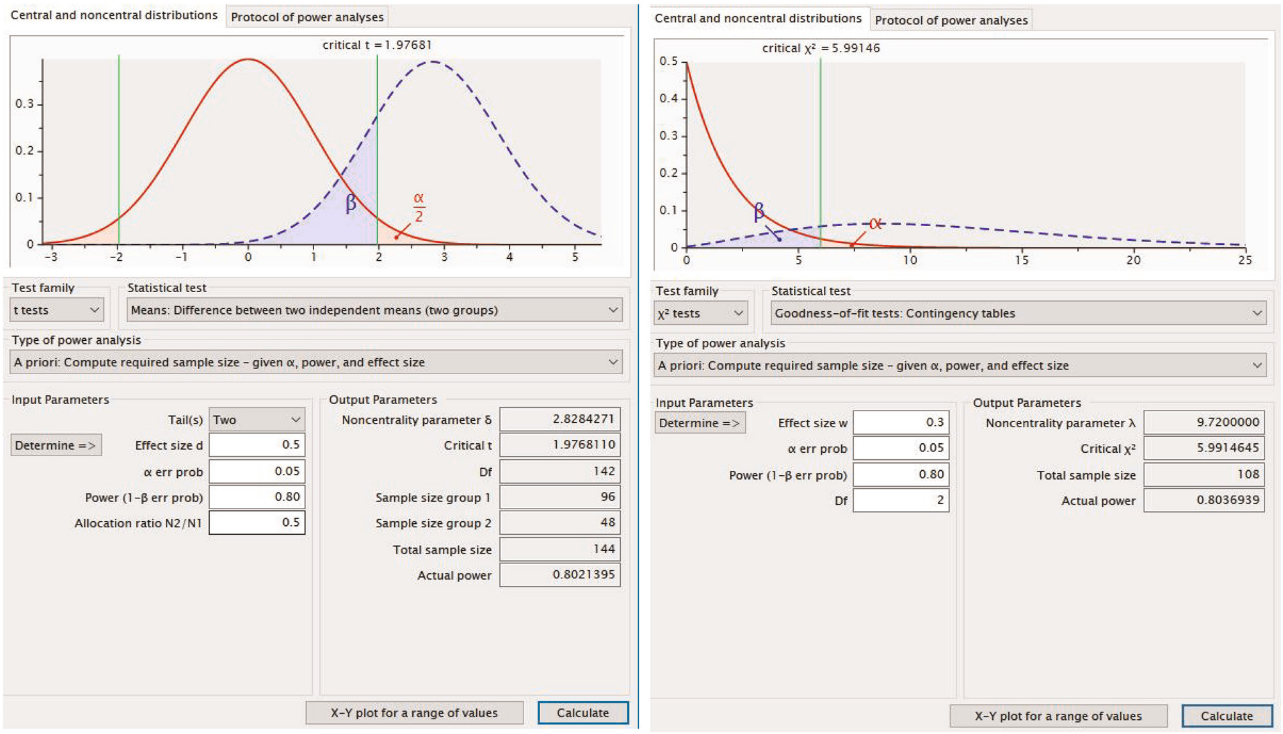

Figure S1: G Power Sample size estimation.
